# Gastrointestinal Microbiota Changes in Patients With Gastric Precancerous Lesions

**DOI:** 10.3389/fcimb.2021.749207

**Published:** 2021-12-09

**Authors:** Dehua Liu, Si Chen, Yawen Gou, Wenyong Yu, Hangcheng Zhou, Rutong Zhang, Jinghao Wang, Fei Ye, Yingling Liu, Baolin Sun, Kaiguang Zhang

**Affiliations:** ^1^ The First Affiliated Hospital of University of Science and Technology of China (USTC), Division of Life Sciences and Medicine, University of Science and Technology of China, Hefei, China; ^2^ School of Life Sciences, University of Science and Technology of China, Hefei, China

**Keywords:** microbiota, *Helicobacter pylori*, gastritis, precancerous lesions, predictive model

## Abstract

**Background:**

Gastric microbiota may be involved in gastric cancer. The relationship between gastrointestinal microbes and the risk of gastric cancer is unclear. This study aimed to explore the gastric and intestinal bacteria associated with gastritis and gastric precancerous lesions. We conducted a case-control study by performing 16S rRNA gene analysis of gastric biopsies, juices, and stool samples from 148 cases with gastritis or gastric precancerous lesions from Anhui and neighboring provinces, China. And we validated our findings in public datasets.

**Results:**

Analysis of microbial sequences revealed decreased bacterial alpha diversity in gastric bacteria during the progression of gastritis. *Helicobacter pylori* was the main contributor to the decreased microbial composition and diversity in the gastric mucosa and had little influence on the microbiota of gastric juice and feces. The gastric mucosal genera *Gemella*, *Veillonella, Streptococcus, Actinobacillus*, and *Hemophilus* had the higher degree of centrality across the progression of gastric precancerous lesions. And *Acinetobacter* may contribute to the occurrence of intraepithelial neoplasia. In addition, the microbial model of *H. pylori*-positive gastric biopsies and feces showed value in the prediction of gastric precancerous lesions.

**Conclusions:**

This study identified associations between gastric precancerous lesions and gastric microbiota, as well as the changes in intestinal microbiota, and explored their values in the prediction of gastric precancerous lesions.

## Highlights

*H. pylori* mainly induces the decreasing diversity and abundance of gastric mucosal microbiota.

The gastric mucosal genera *Streptococcus*, *Acinetobacter*, and *Hemophilus* are related to the progression of gastric precancerous lesions.

Microbial random forest models of *H. pylori*-positive gastric biopsies and stool samples showed performance in the prediction of gastric precancerous lesions.

## Introduction

Gastric cancer (GC) is the fifth most common cancer globally. It is a health threat worldwide and its mortality rate in China ranks third among malignant tumors for men and second for women (Du et al., 2020). Reducing the mortality rate of GC in China is an urgent public health issue (Collaborators, 2020). The key to reducing GC mortality is the early diagnosis of secondary prevention and the identification of high-risk factors related to the occurrence of GC in primary prevention (Bray et al., 2018). The suggested pathogenesis of the intestinal type of non-cardia GC involves development from normal gastric mucosa, erosive gastritis, atrophic gastritis (AG), metaplasia, low-grade neoplasia, and high-grade neoplasia of gastric mucosa to gastric infiltration carcinoma (Correa et al., 1975). However, endoscopic assessment requires experienced endoscopists, and pathological assessment is limited by sampling location when diagnosing precancerous gastric lesions, including atrophic gastritis, intestinal metaplasia (IM), and intraepithelial neoplasia (IN) (Quach and Hiyama, 2019). Therefore, explorations of the etiology and pathogenesis of GC are crucial for early diagnosis and intervention.

Advanced age, male sex, family history, high salt diet, atrophic gastritis, and *H. pylori* infection are general risk factors for GC (Cai et al., 2019). The production of virulence factors, such as cytotoxin-associated gene A and outer membrane protein (Chmiela and Gonciarz, 2017), and the triggering of chronic inflammation are the key carcinogenicity factors of *H. pylori* (Watanabe et al., 2015). The rate of infection by *H. pylori* in China is between 40% and 60%. Only 1% of *H. pylori*-infected individuals subsequently develop malignant gastric tumors (Sugano, 2015). These data indicate that other factors, such as non-*H. pylori* bacteria may be involved in the development of GC (Li and Perez, 2018).

Next-generation sequencing technologies have revealed a close relationship between gastric bacteria other than *H. pylori* and GC (Stewart et al., 2020). Ferreira et al. reported that GC tissues have a unique micro-ecology, in which microbial diversity is reduced compared with healthy gastric mucosa (Ferreira et al., 2018). Several bacterial genera commonly found in the oral cavity, such as *Lactococcus*, *Veillonella*, *Fusobacterium*, and *Leptotrichia*, were found in high abundance in patients with GC (Castano-Rodriguez et al., 2017). *Peptostreptococcus stomatis*, *Streptococcus anginosus*, *Parvimonas micra*, *Slackia exigua*, and *Dialister pneumosintes* are important in the ecological network of GC and inhabit the oral cavity (Coker et al., 2018).

The order *Rhizobiales* was found to be more enriched in patients with IM than in those with superficial gastritis (SG) (Park et al., 2019). Sung et al. have identified that *Acinetobacter lwoffii*, *Streptococcus anginosus*, and *Ralstonia* are associated with persistent inflammation, and that *Granulicatella*, *Actinomyces*, *Rothia*, *Peptostreptococcus*, *Streptococcus*, *Abiotrophia*, and *Parvimonas* are associated with AG or IM in patients successfully treated to eradicate *H. pylori* (Sung et al., 2020). A recent study reported that species belonging to *Streptococcus* and *Hemophilus* were significantly enriched in patients with intraepithelial neoplasia (IN) (Wang et al., 2020). A study indicated that *H. pylori* has a significant effect on the composition of the gastric microbiota, leading to a significant decrease in bacterial diversity (Li et al., 2017). Successful *H. pylori* eradication can lead to the restoration of the gastric microbiota and more beneficial effects on gut microbiota (Guo et al., 2020).

Numerous bacteria detected in gastric biopsies are considered to be part of the oral bacteria because of their significant overlap (Rajilic-Stojanovic et al., 2020). Some common oral bacteria, such as *Neisseria*, *Veillonella*, *Fusobacterium*, *Streptococcus*, and *Hemophilus*, are also enriched in the lower digestive tract (Zoetendal et al., 2012). However, these gastric microbiota associate studies are limited to the changes in the composition of the microbiota between GC and adjacent tissues, or between precancerous lesions and controls biopsies. Overall changes of the bacteria in the gastrointestinal tract during the progression of gastric precancerous lesions (PLGC) have rarely been reported.

We investigated the bacterial microbiota profile in gastric biopsies, juices, and feces for their associations with *H. pylori* and precancerous lesions of gastric carcinoma. We identified gastrointestinal microbes associated with SG, AG, IM, and IN and explored their potential values as PLGC-related biomarkers.

## Materials and Methods

### Patients

We conducted a cross-sectional study of 397 patients with stomach gastritis, residing in Anhui and surrounding provinces, China, who were prospectively recruited between June 2019 and December 2019. Study subjects were recruited from hospital outpatients and completed the 13C-methacetin breath test at the same time. Written informed consent was obtained from each patient. Experienced endoscopists performed all endoscopic examinations and categorizations according to the Kimura-Takemoto classification (Duc et al., 2011), and obtained gastric juices and biopsies using sterile tubes and forceps. Stool samples were collected using sterile sampling tubes before or 2 h after endoscopy. A total of 176 participants completed baseline and follow-up endoscopic examinations and provided gastric biopsies, juices, and stool samples. The patients who used antibiotics and probiotics within 1 month and pump inhibitors within 2 weeks were excluded. General health information of all participants was acquired using a structured questionnaire. This study was approved by the Ethics Committee of the First Affiliated Hospital of University of science and technology of China (2019-ky064, Anhui, China), and informed consent was obtained from all participants before they enrolled in the study.

In addition, we utilized two validation datasets containing 16S rRNA gene sequencing data of 657 gastric biopsies from patients with gastric cancer or precancerous lesions, which were retrieved from the Sequence Read Archive [BioProject: PRJNA375772 (Coker et al., 2018) and PRJEB26931 (Wang et al., 2020)].

### Histological Assessment

Biopsies were taken from the antrum and the gastric body of the participants for microbiota study and histology diagnosis. The biopsied tissues were examined by experienced pathologists. The AG and IM scores were classified into four grades, *i.e.*, 0 = ‘normal’, 1 = ‘mild’, 2 = ‘moderate’, and 3 = ‘marked’, based on the updated Sydney system (Dixon et al., 1996). Scores ≥ 1 were considered positive. IN was classified according to the World Health Organization classification of digestive system tumors and the Vienna classification (Lewin, 1998). Patients with SG were used as controls. Based on the histological diagnosis, we assembled two comparison groups (PLGC vs. SG) to identify PLGC-associated bacteria. The PLGC group was further subdivided into three subgroups (AG, IM, and IN) to understand how gastrointestinal microbiota changes along with the progression of gastritis. We also evaluated the atrophy grade by the OLGA system (Rugge et al., 2007) and IM grade by the OLGIM system (Capelle et al., 2010).

### Sample Sequencing

Two more gastric biopsies were taken from the inflammatory site of the participants’ gastric antrum and body for microbial detection. All gastric biopsies, juices, and stool samples were frozen immediately after collection within 0.5 h and stored at -80°C until analysis. DNA was extracted from gastric biopsy and juice samples using the low-salt CTAB method (Arseneau et al., 2017) and from stool samples using the E.Z.N.A. ^®^Stool DNA Kit (D4015; Omega Bio-Tek, Norcross, GA, USA), according to the manufacturer’s instructions. Total DNA was eluted in 50 μl of elution buffer and stored at -80°C until measurement by LC-Bio Technology Co., Ltd, (Hang Zhou, China). Purification of DNA for the bacterial small subunit (16S) rRNA gene sequencing was performed on a total of 528 samples. Of these, 246 samples were excluded due to failed DNA extraction and exclusion criteria, and sequencing was completed for 66 gastric biopsies, 68 gastric juices, and 148 stool samples. The V3-V4 region of the 16S rRNA gene was amplified with primers 341F (5’-CCTACGGGNGGCWGCAG-3’) and 805R (5’-GACTACHVGGGTATCTAATCC-3’) (Logue et al., 2016). Amplicon pools were prepared for sequencing and the size and quantity of the amplicon library were assessed on a 2100 Bioanalyzer system (Agilent, Santa Clara, USA) and with a Library Quantification Kit for Illumina (Kapa Biosciences, Woburn, MA, USA), respectively. The libraries were sequenced on a NovaSeq PE250 platform.

### Sequence Curation and Annotation

Sequencing quality filtering and analysis were performed using the QIIME2 pipeline (v2020.11) (Bolyen et al., 2019). Paired-end reads were filtered, merged, and dereplicated using VSEARCH and the Deblur plugin, which reduced the feature table and feature sequences (the “features” resulting from Deblur are created by grouping unique sequences). The samples with > 10000 frequency were reserved for subsequent analysis, including public datasets. The SILVA 16S database (v132) was used for taxonomy assignment of sequence datasets and performed by the QIIME2 plugin feature classifier. The metagenome functional profiling of the gastrointestinal microbial communities was estimated by the Phylogenetic Investigation of Communities by Reconstruction of Unobserved States-PICRUSt2 (v2.0.0) (Douglas et al., 2020). Differentially pathways with a false discovery rate adjusted *p*-value <0.05 were presented.

### Statistical Analyses

Alpha diversity analysis was performed using the QIIME2 process and illustrated by Faith’s phylogenetic diversity, Chao1, and Shannon indexes, which were assessed using an ANOVA test for multiple groups. Multiple group comparisons of dissimilarities between groups were made using the permutational multivariate analysis of variance test. Microbial diversity was visualized using the discriminant analysis of principal components based on the pathological diagnosis group. Taxonomic discovery analysis was performed using R package DESeq2 and linear discriminant analysis effect size (LEfSe, http://huttenhower.sph.harvard.edu/galaxy/), and bacteria with > 0.1% relative abundance (RA) were exhibited (Segata et al., 2011). DESeq2 was used in combined public datasets with cohort information added to adjust the batch effect. The identification of co-occurring and co-exclusion bacteria at the genus level was estimated using the SparCC algorithm (Coker et al., 2018), and visualized by Gephi (v0.9.2) (Heymann and Grand, 2013). Network parameters including topological coefficient, closeness, and betweenness were estimated using igraph (v1.2.5) and compared using the Wilcoxon test. Data visualization was performed by the R Project (v4.0.2). Univariate analysis of functional features between multiple diagnosis groups was undertaken with an ANOVA test using STAMP (v2.1.3) (Parks et al., 2014). All *p* values < 0.05 after multiple comparisons correction using the false discovery rate method were considered significantly different.

## Results

### Patient Demographics and Assessment

The data presented pertain to samples from a total of 148 subjects (148 stool samples, 66 gastric biopsies, and 68 gastric juices) after exclusion and successful sequencing (**Supplementary Figure 1**). The baseline characteristics of participants and public data are presented in **Supplementary Tables 1, 2**. We performed endoscopic diagnosis according to the Kimura-Takemoto classification (Duc et al., 2011) and further pathological diagnosis according to the updated Sydney system (Dixon et al., 1996), operative link on gastritis assessment (OLGA) staging system (Rugge et al., 2007), and the modified operative links of gastric intestinal metaplasia (OLGIM) system (Capelle et al., 2010) (**Supplementary Figures 2A, C**). The histological assessment of AG showed good agreement with the Kimura-Takemoto classification with a moderate correlation (**Supplementary Figure 2B**).

The participants were tested for *H. pylori* infection using 13C-UBT. Mucosal biopsies with >1% *H. pylori* RA were classified as *H. pylori*-infected, the consistency between the conventional detection methods and the results based on 16S rRNA gene sequencing was only 78.94% in our dataset, and 49.02% in validation datasets (**Supplementary Table 3**). The participants were grouped as *H. pylori*-positive in biopsies with >1% *H. pylori* RA, whereas biopsies samples with <1% *H. pylori* RA were grouped as *H. pylori*-negative in all the datasets as previously described (Kim et al., 2015).

### *H. pylori* Decrease the Diversity of Gastric Mucosa Microbiota

**Figure 1:**
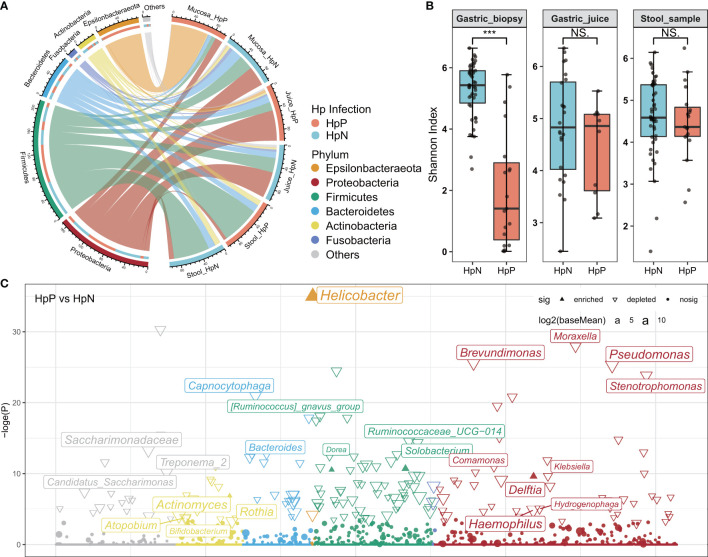
The influence of *H. pylori* on gastrointestinal microbiota composition and diversity. **(A)** The abundance of the gastrointestinal microbiota is shown at the phylum level. **(B)** Bacterial diversity was estimated by the Shannon index for HpP and HpN groups. **(C)** Association of specific bacteria taxa with *H. pylori* infection was identified by DESeq2 with adjusted *p* < 0.05. HpP, *H. pylori*-positive; HpN, *H. pylori*-negative; NS, not significant, ***p < 0.001.

The overall RA at the phylum level indicated less diverse bacterial taxa among gastric biopsies in the *H. pylori*-positive group (**Figure 1A**). The taxonomic composition of the mucosa of *H. pylori-*infected participants compared with *H. pylori*-negative subjects included significantly increased Epsilonbacteraeota (mean RA from 0.91% to 68.22%, adjusted *p* < 0.001) and significantly decreased Firmicutes (mean RA from 27.55% to 8.18%, adjusted *p* < 0.01), Proteobacteria (mean RA from 36.53% to 13.97%, adjusted *p* < 0.01), and Bacteroidetes (mean RA from 19.70% to 6.16%, adjusted *p* < 0.05). However, there was no significant change in the proportion of Epsilonbacteraeota and other phyla in the stool and gastric juice samples of the *H. pylori*-positive group compared with the *H. pylori*-negative group. The Shannon index of alpha diversity analysis showed a lower bacterial diversity in biopsies of the *H. pylori*-positive group (Wilcoxon test, *p* < 0.001), and no significant change in gastric juices and stool samples (**Figure 1B**). We also observed that the *H. pylori* RA is negatively correlated with mucosal bacterial diversity in validation data (**Supplementary Figure 3**). Beta diversity analysis with PCoA based on the feature level revealed a significant difference in gastric mucosal microbiota between the *H. pylori*-positive group and *H. pylori*-negative group (**Supplementary Figure 4**).

Biomarker discovery using the R package DESeq2 showed that *Helicobacter* was enriched in the *H. pylori*-positive group of gastric biopsies, whereas most genera were depleted (RA > 0.1%, **Figure 1C**). In gastric juice and stool samples, there were few changes in bacteria diversity, composition, and genera between *H. pylori*-positive and *H. pylori*-negative groups. A heatmap was constructed using data from the top 30 genera among all gastrointestinal samples (**Supplementary Figure 5**). The high abundance taxa, including *Streptococcus, Hemophilus*, and *Veillonella* were dominant in most samples. *Halomonas* and *Shewanella* were dominant in gastric biopsies, *Sphingomonas*, *Acinetobacter*, and *Curvibacter* were enriched in gastric juices, while *Bacteroides* and *Faecalibacterium* were mainly in stool samples. The high abundance of *Helicobacter* was detected in all gastric biopsies, in some of the juice samples, and none of the stool samples of the *H. pylori*-positive group. *Helicobacter* was also detected at low levels in the stomach and intestine of some *H. pylori*-negative subjects.

### Non-*H. pylori* Bacterial Diversity Decreases in Gastric Mucosa of Subjects With Gastric Precancerous Lesions

**Figure 2:**
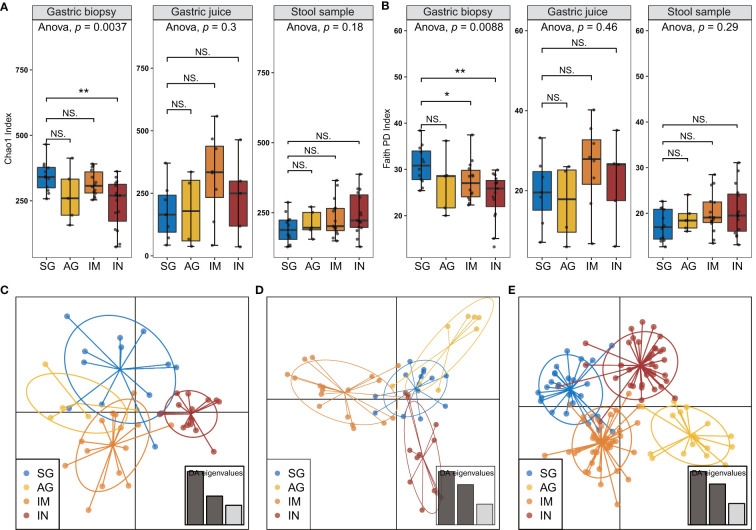
Microbial diversity and community structure in gastric biopsies, juices, and stool samples of *H. pylori-*negative subjects. Alpha diversity was estimated by **(A)** Chao1 and **(B)** Faith’s phylogenetic diversity indexes for diagnosis groups. The discriminant analysis of the principal components (DAPC) plot at the features level shows distinct clustering of the diagnosis groups in **(C)** gastric biopsies, **(D)** juices, and **(E)** stool samples. *adjusted *p* < 0.05. NS, not significant, **p < 0.01.

*H. pylori-*positive subjects were excluded from the disease-associated bacterial diversity analysis because of the significantly reduced microbial diversity induced by *H. pylori* infection, according to our findings and previous studies (Alarcon et al., 2017; Li et al., 2017). The alpha diversity of gastric mucosal bacteria, including the Chao1 and Faith’s phylogenetic diversity indexes, showed a significant decrease in the IN group compared with the SG group at the features level (**Figures 2A, B**). The decreasing trend in Faith’s phylogenetic diversity index of gastric mucosal microbiota was also found across the different stages of OLGA and OLGIM (**Supplementary Figure 6**). We also observed that phylogenetic diversity of mucosal bacteria significantly decreased in patients with IN compared with SG, AG, and IM in validation data (**Supplementary Figure 7**). The collective results suggested the disorder of gastric mucosal microbiota in subjects with gastric precancerous lesions, especially in the stage of intraepithelial neoplasia. However, there was no difference observed in gastric juices and fecal microbial diversity between the different diagnosis groups.

Differences in microbial community structure were further evaluated using discriminant analysis of principal components analysis (Jombart et al., 2010). The analysis separated each histological diagnosis group into different clusters in all the samples (**Figures 2C–E**). Beta diversity analysis using the Weighted-Unifrac distance matrices of gastrointestinal samples was conducted to assess dissimilarities among all groups. And the results showed that only the mucosal microbiota had significant differences between diagnosis groups (**Supplementary Figure 8**).

### Gastrointestinal Microbiota Ecology Is Altered in Gastric Precancerous Lesions

**Figure 3:**
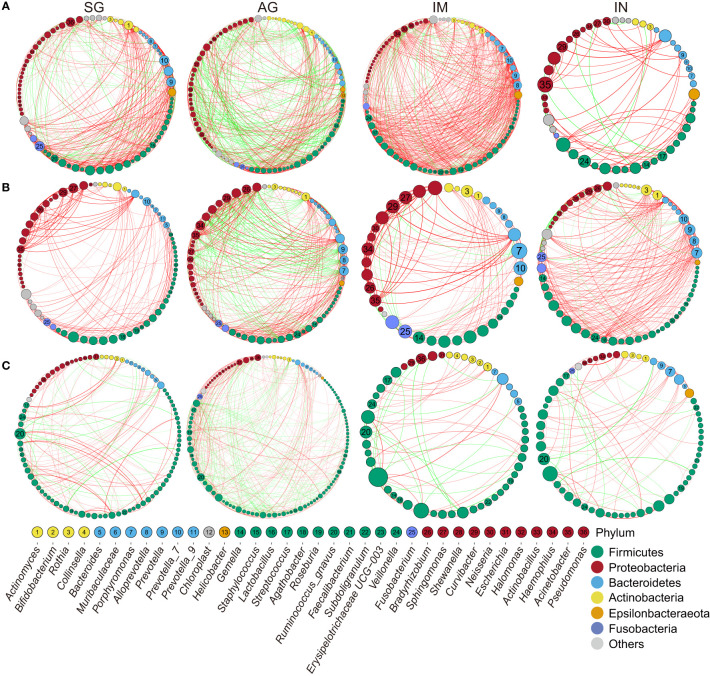
Correlation networks of the gastrointestinal genus in different PLGC groups. **(A)** The interactions of bacteria in gastric biopsies in *H. pylori*-negative subjects with strengths > 0.8. **(B)** The interactions of bacteria in gastric juice with strengths > 0.8. **(C)** The interactions of bacteria in stool samples with strengths >0.5. The size of nodes corresponds to weighted node connectivity scores, and the nodes were colored by phylum. Red edges denote positive correlations and green edges denote negative correlations.

We performed a microbial co-occurrence and co-exclusion network and topology analysis at different stages of gastritis and PLGC to explore the interplay of the gastrointestinal microbiota. Overall, the strength of co-occurring interactions among genera increased in IM, and decreased in IN of *H. pylori*-negative gastric biopsies (**Figure 3A**). We observed that the genera *Gemella*, *Veillonella, Streptococcus, Actinobacillus*, and *Hemophilus* had the higher degree of centrality and strong co-occurrence interaction in gastric biopsies across the PLGC stages. And *Acinetobacter* co-occurred with a variety of genera in IN. The similar co-occurring interactions trend and central bacteria in each PLGC subgroup of *H. pylori*-negative gastric biopsies was also observed in validation datasets (**Supplementary Figure 9**). The co-occurrence interactions between *Curvibacter*, *Sphingomonas*, *Acinetobacter*, and *Fusobacterium* were observed in gastric juices (**Figure 3B**). In stool samples, several genera of Firmicutes, such as *Ruminococcus gnavus* and *Veillonella*, exhibited a high degree of centrality (**Figure 3C**).

### Gastrointestinal Bacteria Associated With Gastric Precancerous Lesions

**Figure 4:**
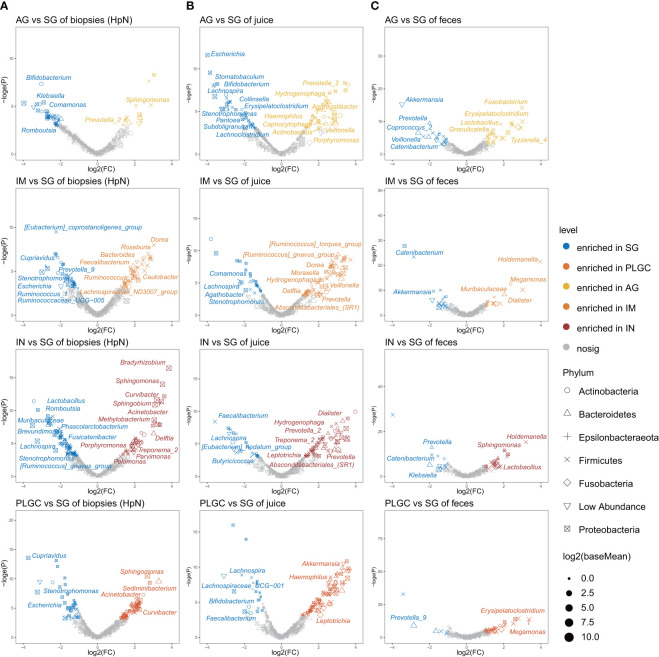
Specific bacterial taxa associated with PLGC. **(A)** Significantly changed mucosal bacteria in different PLGC subgroups of *H. pylori*-negative subjects. **(B)** Significantly changed bacteria in diagnosis groups of gastric juices and **(C)** stool samples.

We then assessed if the bacteria could potentially contribute to gastric precancerous lesions in patients using DESeq2. The results showed that *Prevotella_2* and *Sphingomonas* were enriched in the AG group, *Dorea*, *Caulobacter*, and *Bacteroides* were enriched in the IM group, and *Bradyrhizobium*, *Sphingomonas*, *Curvibacter*, and *Acinetobacter* were enriched in the IN group of *H. pylori*-negative biopsies (**Figure 4A**). Differential bacteria analysis using the LEfSe test between diagnosis groups was also performed. Enriched *Bifidobacterium* and *Klebasiella* in SG, and enriched *Sphingomonas* and *Acinetobacter* in IN were observed in gastric biopsies (**Supplementary Figure 10**). In validation datasets, we found that *Acinetobacter* was enriched in the IN group of both *H. pylori*-positive and *H. pylori*-negative biopsies (**Supplementary Figure 11**). Enriched *Prevotella_2* in AG and enriched *Dorea* in IM were observed in gastric juices (**Figure 4B**). The significantly changed genera in AG and IM groups of biopsies may be influenced by the changing bacteria in gastric juice. In stool samples, *Akkermmansia* and *Catenibacterium* in SG, *Lactobacillus* in AG and IN, and *Holdemanella* in IM and IN were the enriched genera (**Figure 4C**).

### The Value of Gastrointestinal Microbiota in Predicting the Risk of Gastric Cancer

**Figure 5:**
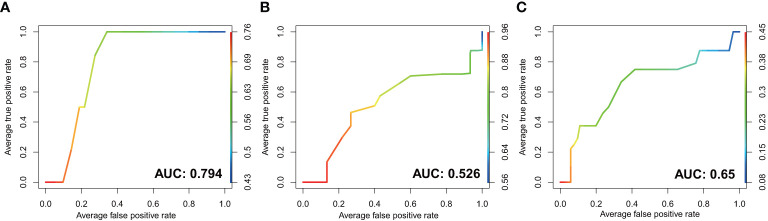
The performance of GI microbiota in prediction of PLGC by receiver operating characteristic (ROC) curve analysis. The discriminatory potential of the mucosal microbial model in **(A)** *H. pylori*-positive subjects and **(B)** *H. pylori*-negative subjects. **(C)** The discriminatory potential of the microbial model in stool samples.

We further constructed random forest models based on the mucosal bacteria with > 0.1% RA in combined validation datasets and tested them in our dataset, to assess the value of gastrointestinal microbiota in predicting the risk of gastric cancer. The model of mucosal microbiota showed excellent performance in distinguishing PLGC and SG with an AUC of 0.794 in *H. pylori*-positive subjects (**Figure 5A**). Whereas the model of mucosal microbiome in *H. pylori*-negative subjects yielding an AUC of 0.526 (**Figure 5B**). Additionally, the model based on intestinal microbiota distinguished PLGC from SG with an AUC of 0.65 in our dataset (**Figure 5C**). The important genera that contributed to the models were selected by R package Boruta and listed in **Supplementary Table 4** (Miron and Kursa, 2010).

### The Functions of Mucosal Microbiota Changed in Patients With PLGC

The functional predictions in combined datasets that identified KEGG orthologs, Enzyme Classification, and metaCyc pathways were performed using PICRUSt2 (**Supplementary Table 5**). The superpathway of N-acetylglucosamine (GLCMANNANAUT-PWY), superpathway of (Kdo)2-lipid A biosynthesis (KDO-NAGLIPASYN-PWY), superpathway of N-acetylneuraminate degradation (P441-PWY), superpathway of demethylmenaquinol-8 biosynthesis (PWY-5861), and Ribonucleoside-triphosphate reductase (EC:1.17.4.2) were found significantly enriched in the IN group of both *H. pylori*-positive and *H. pylori*-negative subjects. No significantly changed function was found in gastric juices and stool samples between multiple diagnosis groups.

## Discussion

We simultaneously examined the bacteria of gastric mucosa, juice, and feces in different stages of gastric precancerous lesions, and validated our findings in two public datasets, to explore the PLGC-associated bacteria. Different gastrointestinal microbiota profiles were evident between the gastritis diagnosis groups. The major findings revealed changed gastrointestinal microbial diversity and interaction across the stages of gastric precancerous lesions, especially in the stage of IN. We also identified that the genus *Gemella*, *Streptococcus*, *Actinobacillus*, *Hemophilus*, and *Acinetobacter* were associated with the development of PLGC.

A previous study reported that the critical RA of the *H. pylori* colonization rate in conventional methods that identified *H. pylori*-positive subjects was about 1.22% (Kim et al., 2015). In our study, the consistency between the positive rate of the high-throughput assay and positive 13C-UBT results was less than 75%, when the biopsies with >1% *H. pylori* RA were grouped as *H. pylori*-positive. A low proportion (RA < 0.1%) of *H. pylori* was found in some gastric biopsies, juices, and stool samples of *H. pylori*-negative subjects. The presence of *H. pylori* in the gastric biopsies of several patients who were deemed *H. pylori*-negative by conventional diagnostic testing was also reported in several previous studies (Bik et al., 2006; Kienesberger et al., 2016; Li et al., 2017). These findings suggested that the conventional diagnostic tests may underestimate the prevalence of *H. pylori* infection in the population. Some *H. pylori*-negative individuals had *H. pylori* sequences in their stool samples as well, which may be the result of host resistance to *H. pylori* infection. We also found that the *H. pylori* sequence was not detected in gastric juices and stool samples of some *H. pylori*-positive individuals, suggesting that *H. pylori* is not the dominant species in the gastric juice and feces. Analysis of alpha diversity showed a decreased microbial diversity according to the Shannon index in *H. pylori*-positive subjects, confirming previous studies (Bik et al., 2006; Parsons et al., 2017), whereas there was no significant difference in gastric juice and stool samples. These results suggest that *H. pylori* mainly influences the microbial composition and diversity in gastric mucosa.

Our network analysis showed an increased strength of co-occurring interactions among genera in IM which then decreased in IN of gastric biopsies with gastritis progression. A previous study also observed that the co-occurrence interactions were stronger in IM than SG (Coker et al., 2018). These suggested that the bacteria tend to co-occur to form a specific microecology prior to the occurrence of neoplasia. *Gemella*, *Veillonella, Streptococcus, Actinobacillus*, and *Hemophilus* had higher degrees of centrality in all gastric biopsies, and *Curvibacter*, *Sphingomonas*, *Acinetobacter*, and *Fusobacterium* had higher degrees of centrality across PLGC groups in gastric juices. In addition, *Acinetobacter* had the highest degree of centrality in gastric biopsies of the IN group. The microecological network formed by these genera may be related to the occurrence and development of PLGC. *Gemella*, *Veillonella*, and *Streptococcus* were reported to be the predominant genera in the upper gastrointestinal tract (Vasapolli et al., 2019). A previous study identified that *Acinetobacter lwoffii* and *Streptococcus anginosus* were associated with persistent gastric inflammation (Sung et al., 2020). Whether the gastritis-associated *Acinetobacter* was colonized in gastric mucosa or derived from gastric juice still needs further proof.

Alpha diversity analysis showed a decreasing Faith’s phylogenetic diversity index in the development of PLGC, and a similar tendency was identified in the validation datasets. The significantly reduced mucosal microbiota diversity in GC compared with chronic gastritis was reported in several studies (Guo et al., 2020; Stewart et al., 2020). We further demonstrated that the diversity and composition of gastric mucosa microbiota have already been significantly changed during the pre-cancerous intraepithelial neoplasia stage.

To identify the bacteria that may potentially contribute to the progression of PLGC, we further compared the bacteria between different groups. In the gastric mucosa of *H. pylori*-negative subjects, *Prevotella_2* and *Sphingomonas* were enriched in AG, *Dorea*, *Caulobacter*, and *Bacteroides* were enriched in IM, and *Bradyrhizobium*, *Sphingomonas*, *Curvibacter*, and *Acinetobacter* were enriched in IN. Compared with SG, the significantly changed bacteria in each subgroup of PLGC were quite different. The enriched *Acinetobacter* in mucosal dysplasia was also identified in a previous study and public datasets (Kadeerhan et al., 2021). Whereas Wang et al. reported that *Streptococcus*, *Neisseriaceae*, and *Hemophilus parainfluenza* were significantly enriched in IN (Wang et al., 2020).

In addition, functional pathways related to (Kdo)2-lipid A biosynthesis, N-acetylneuraminate degradation, and demethylmenaquinol-8 biosynthesis were altered in IN. (Kdo)2-lipid A is the essential component of lipopolysaccharides in most Gram-negative bacteria, which may stimulate potent host immune responses through the complex of Toll-like-receptor 4 (TLR4) (Wang et al., 2015). Demethylmenaquinol was reported to be a substrate of *Escherichia coli* nitrate reductase A, which may contribute to nitrite production (Rendon et al., 2015). However, the role of these significantly changed bacteria and functional pathways in the occurrence and development of PLGC still needs further study.

Several decreased genera in the PLGC group of gastric juices and stool samples were also observed. The changes in gastric juice bacteria between diagnosis groups were not consistent with mucosal microbiota, indicating that the PLGC-related bacteria in biopsies were not affected by oral or esophageal microbiota. In the *H. pylori*-positive subjects, the number of identified significantly changed genera between PLGC groups was much more than that in *H. pylori*-negative subjects, whether these bacteria have synergistic proinflammatory effects with *H. pylori* remains to be discussed.

Few studies had identified that the significantly changed non-*H. pylori* genera could be used as the potential microbial biomarkers for GC and precancerous lesions (Coker et al., 2018; Wang et al., 2020; Kadeerhan et al., 2021). However, we found that the excellent performance of the gastric mucosal microbial model to predict PLGC was only shown in *H. pylori*-positive subjects. It suggested that the influence of *H. pylori* should be fully considered in the study of these GC/PLGC-associated microbiota. The random forest model of fecal microbiota also exhibited a value to discriminate PLGC from SG, with little or no influence from *H. pylori*.

Our study still has several limitations. There was a lack of gastric microbiota of GC patients in our study. Although OLGA and OLGIM staging were performed, there was a lack of analysis on the atrophy and intestinal metaplasia-related microbiota due to the small number of subjects in stages III and IV. And there were fewer gastric juice samples and gastric biopsies compared to stool samples because of failed DNA extraction and sequencing. Our findings on gastric mucosa bacteria were validated in public datasets, but the changes of gastric juice and fecal microbiota were less validated. In addition, our study was mainly based on the Chinese population and did not address the influence of factors such as smoking and alcohol consumption on the GI microbiota.

In conclusion, our study demonstrated the changes of gastrointestinal microbiota across the progression of gastric precancerous lesions. The diversity and interactions of gastric mucosal bacteria significantly decreased in the stage of intraepithelial neoplasia. The differences of gastric precancerous lesion-associated bacteria and functional pathways of *H. pylori*-negative subjects were also observed. Microbiome models of *H. pylori*-positive gastric biopsies and stool samples showed potential in the prediction of PLGC. Subsequent confirmatory experimental studies in a broader population are further needed to examine whether these bacteria contribute to the precancerous progression of GC.

## Data Availability Statement

The datasets presented in this study can be found in online repositories. The names of the repository/repositories and accession number(s) can be found in the article/**Supplementary Material**.

## Ethics Statement

This study was approved by the Ethics Committee of the First Affiliated Hospital of University of science and technology of China (2019-ky064, Anhui, China), and informed consent was obtained from all participants before they enrolled in the study. The patients/participants provided their written informed consent to participate in this study.

## Author Contributions

KZ designed and supervised this study. SC, YG, FY, and YL conducted upper endoscopy examinations. KZ and SC contributed to subject recruitment and DL, RZ, and JW contributed to sample collection. HZ and WY completed histopathological diagnoses. DL analyzed the experimental results and wrote the first draft of the manuscript. KZ and BS revised the manuscript. All authors contributed to the article and approved the submitted version.

## Funding

This work was supported by the Science and Technology project of Anhui under grant 1604a0802075.

## Conflict of Interest

The authors declare that the research was conducted in the absence of any commercial or financial relationships that could be construed as a potential conflict of interest.

## Publisher’s Note

All claims expressed in this article are solely those of the authors and do not necessarily represent those of their affiliated organizations, or those of the publisher, the editors and the reviewers. Any product that may be evaluated in this article, or claim that may be made by its manufacturer, is not guaranteed or endorsed by the publisher.
