# Supplementary material for: Gastrointestinal Microbiota Changes in Patients With Gastric Precancerous Lesions
Source: Front Cell Infect Microbiol. 2021 Dec 9;11:749207. doi: 10.3389/fcimb.2021.749207 (PMC8695999; doi:10.3389/fcimb.2021.749207)
Supplement: Supplementary file 2 [file DataSheet_2.docx]

Supplementary Figures

**
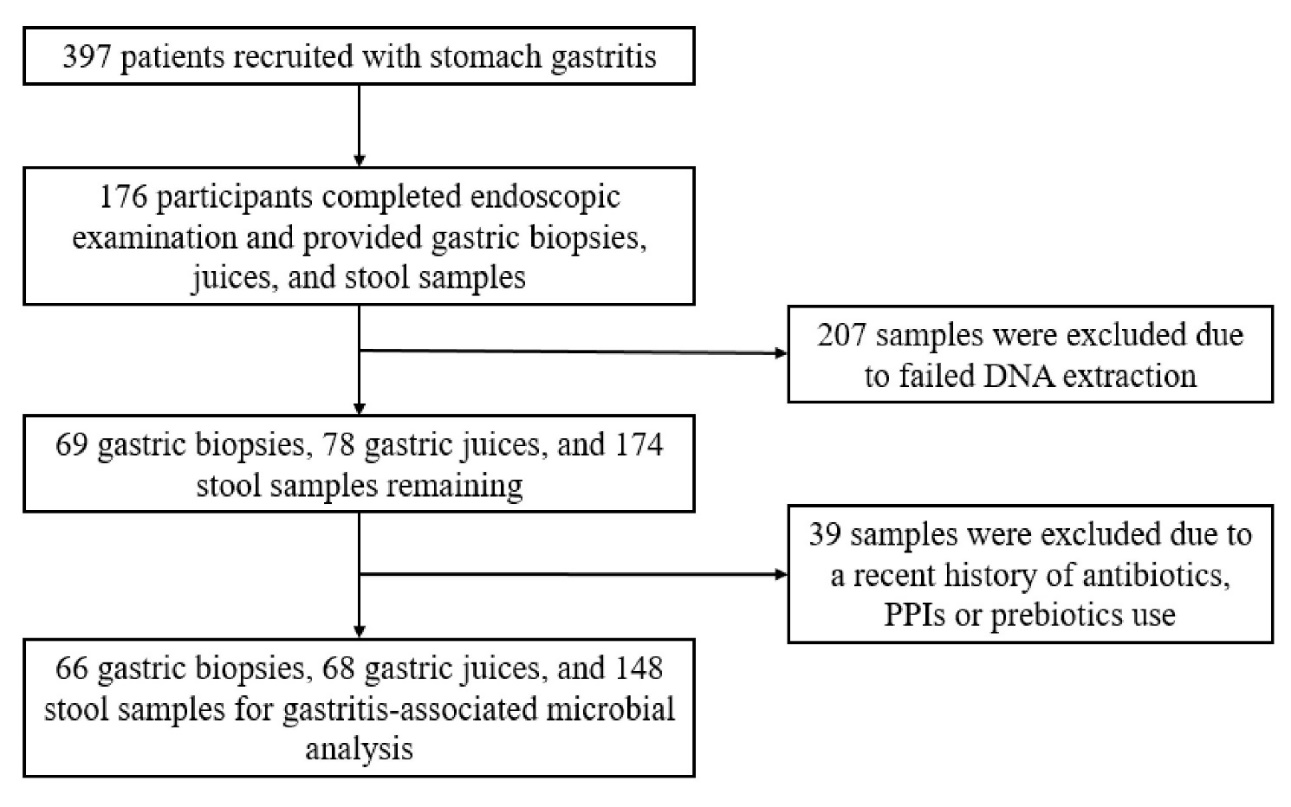
**

**Figure S1.** Sample selection flow diagram.


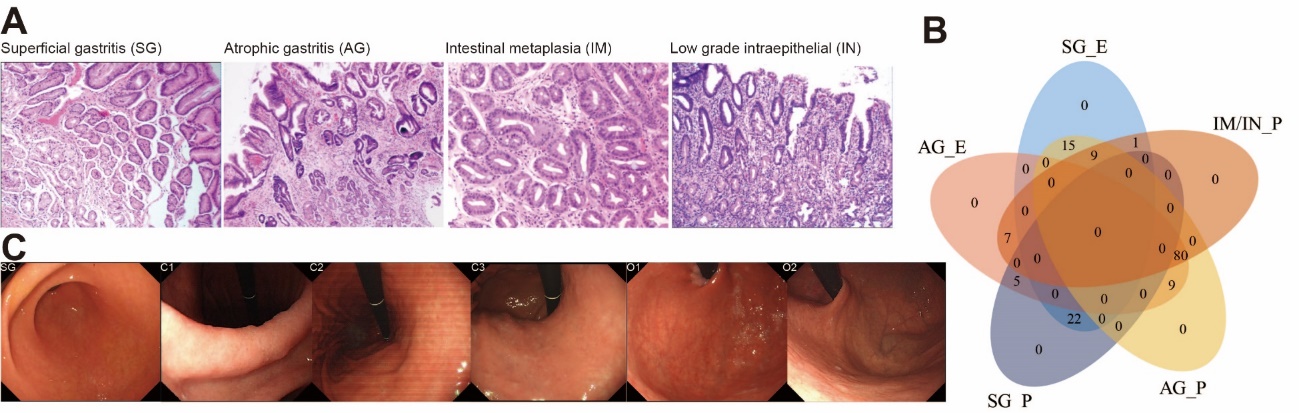


**Figure S****2.** Comparison of gastroscopy and pathology diagnosis of participants. (A) Pathological diagnosis results according to the updated Sydney system. (B) Different diagnosis results between endoscopic and pathological assessment. (C) Endoscopic results according to the Kimura-Takemoto classification system. SG: superficial gastritis, AG: atrophic gastritis, IM: intestinal metaplasia, IN: intraepithelial neoplasia, E: Endoscopic diagnosis, P: pathologic diagnosis.

*
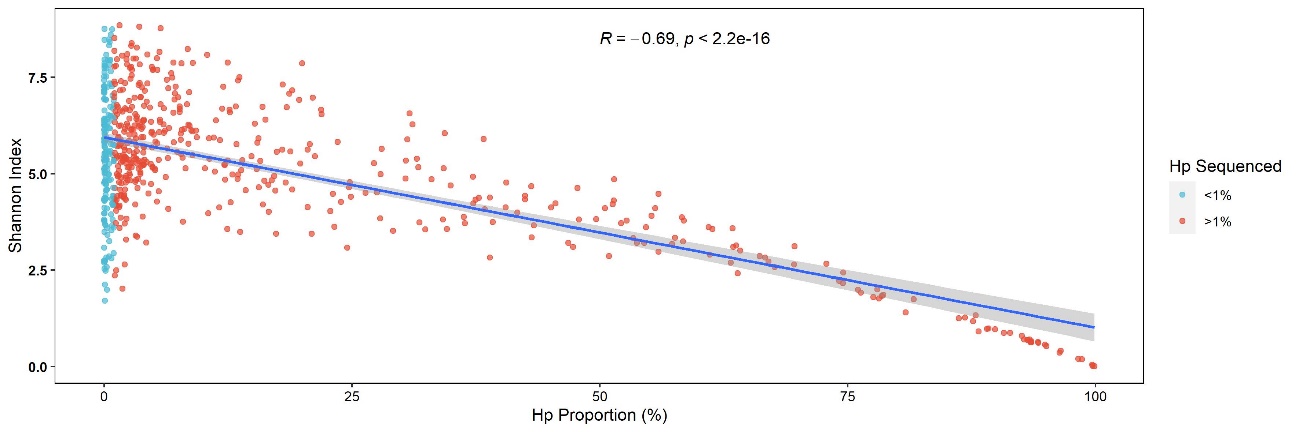
*

**Figure S3.** The correlation between *H. pylori* relative abundance and Shannon index in gastric biopsies of validation datasets.


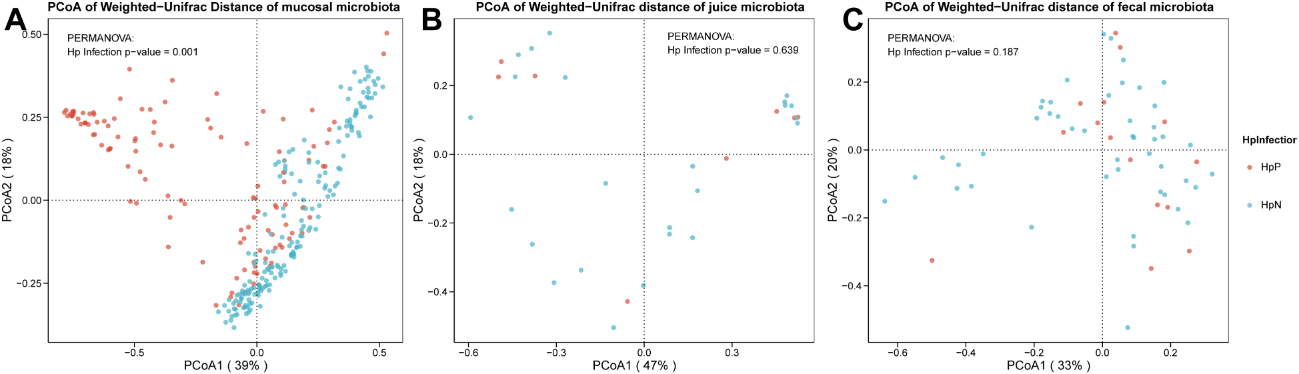


**Figure S****4.** Microbial community structure in gastrointestinal samples. (A) Principal coordinate analysis (PCoA) plots and permutational multivariate analysis of variance (PERMANOVA) test based on Weighted-Unicrac distance for *H. pylori*-positive group and *H. pylori* -negative group in (A) gastric biopsies, (B) gastric juices, and (C) stool samples.


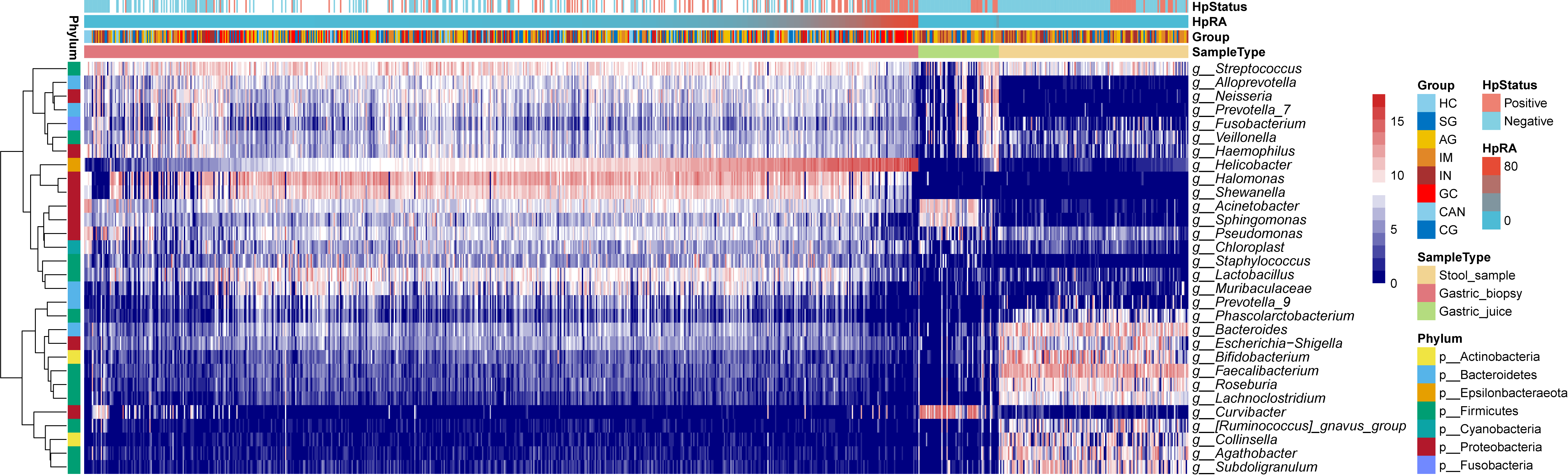


**Figure S****5.** Top 30 genera in gastrointestinal of all subjects with gastritis.


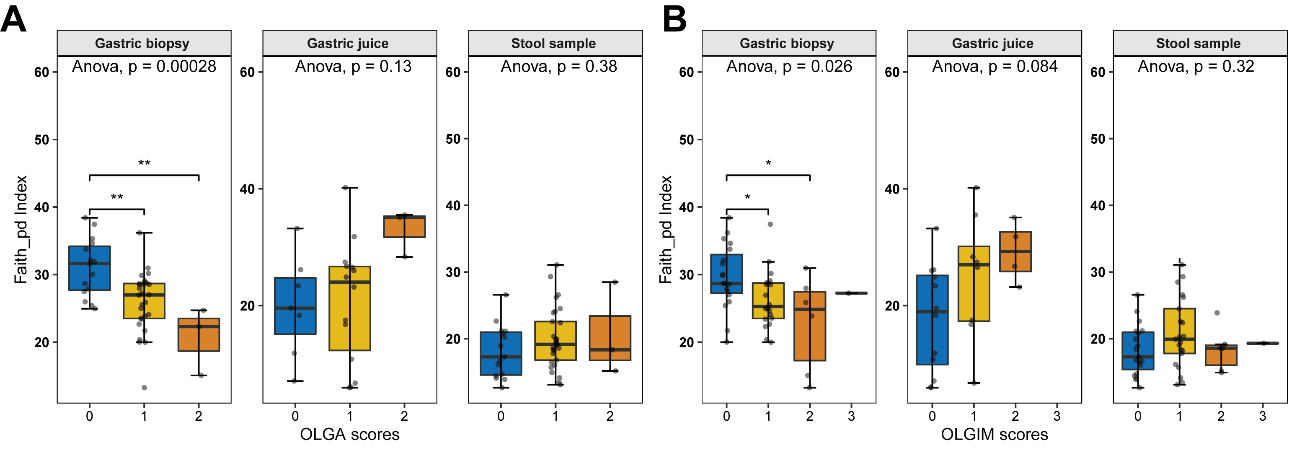


**Figure S****6.** Microbial diversity in gastric biopsies, juices, and stool samples of *H. pylori*-negative subjects in self-sequenced dataset. Alpha diversity was estimated with Faith's phylogenetic diversity index for (A) OLGA and (B) OLGIM system. *adjusted *p* < 0.05, ** adjusted *p* < 0.01.


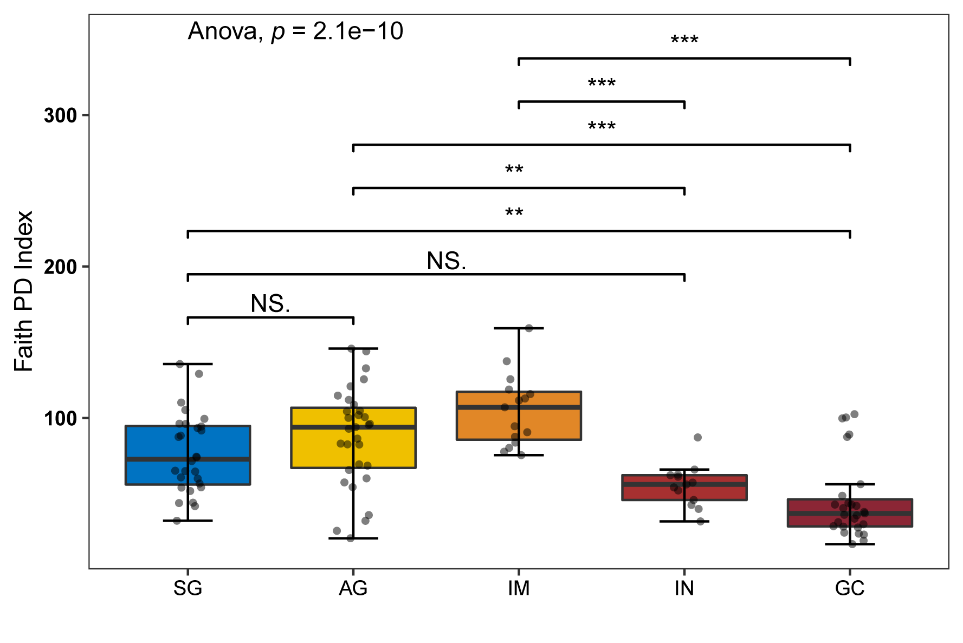


**Figure S****7.** Microbial alpha diversity in *H. pylori*-negative gastric biopsies of validation datasets. *adjusted *p* < 0.05, ** adjusted *p* < 0.01, *** adjusted *p* < 0.001.


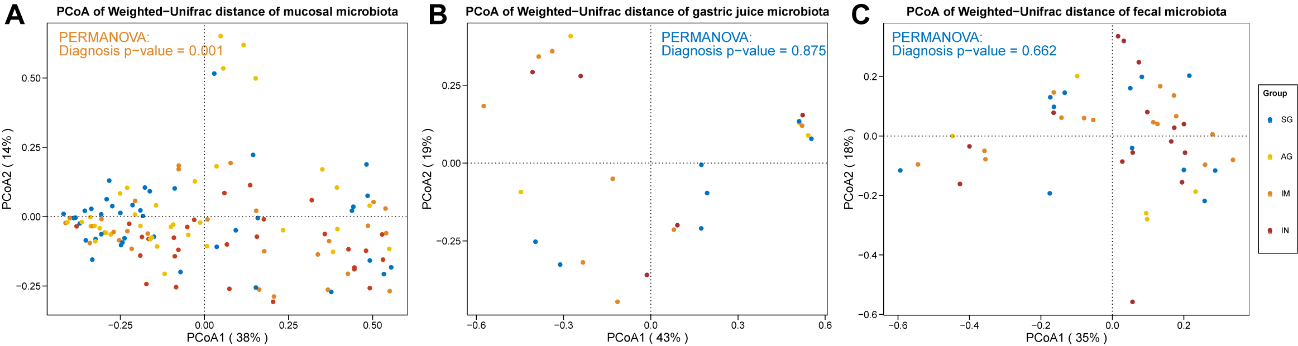


**Figure S****8.** Microbial community structure in gastrointestinal samples of *H. pylori*-negative subjects. PCoA plots and PERMANOVA test based on Weighted-Unicrac distance for diagnosis groups in (A) gastric biopsies, (B) gastric juices, and (C) stool samples.


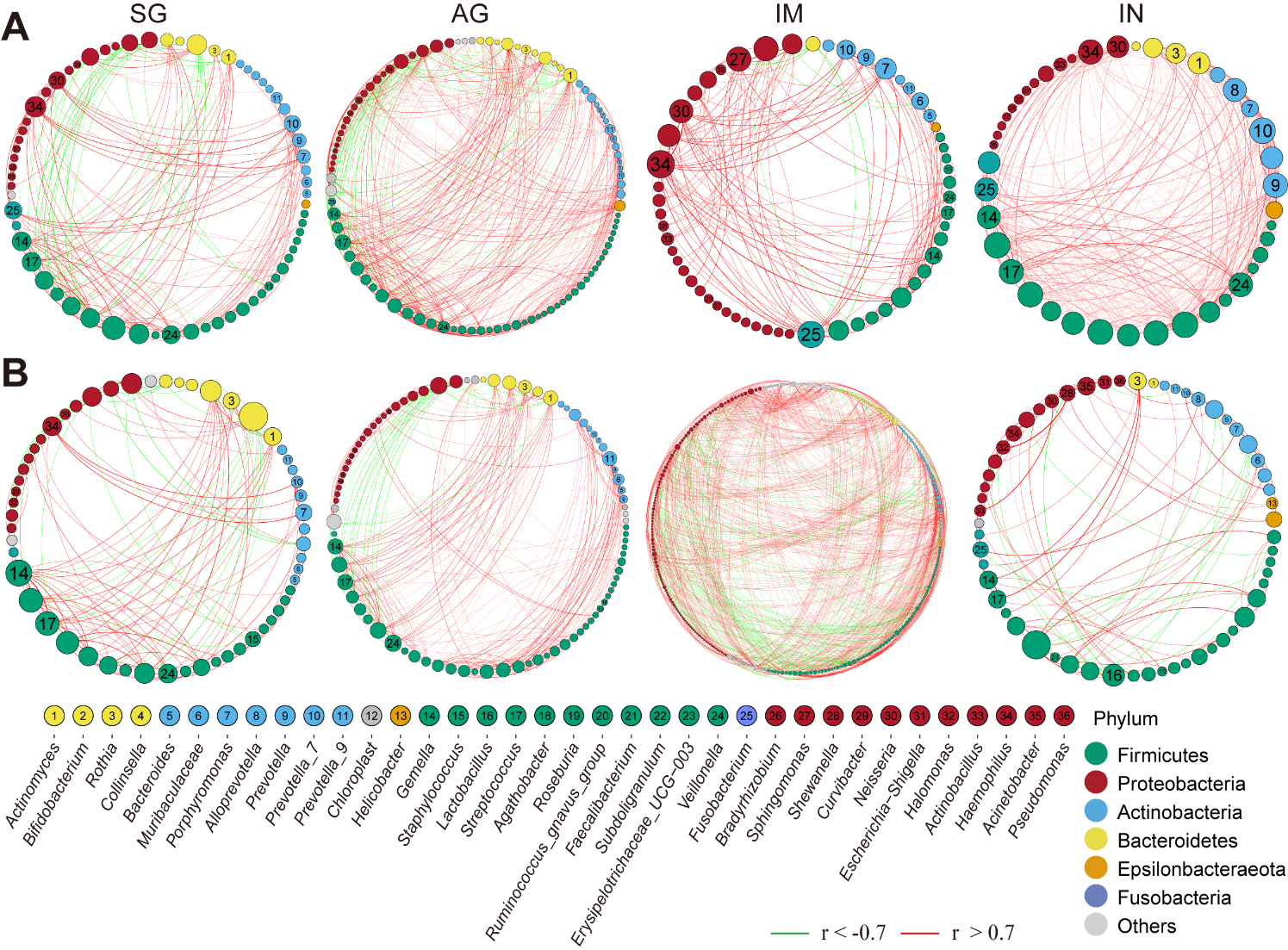


**Figure S9.** Correlation networks of the gastrointestinal genus in different PLGC groups of validation datasets. (A) The interactions of bacteria in gastric biopsies in *H. pylori*-positive subjects with strengths > 0.7. (B) The interactions of bacteria in gastric biopsies in *H. pylori*-negative with strengths > 0.7. The size of nodes corresponds to weighted node connectivity scores, and the nodes were colored by phylum. Red edges denote positive correlations and green edges denote negative correlations.


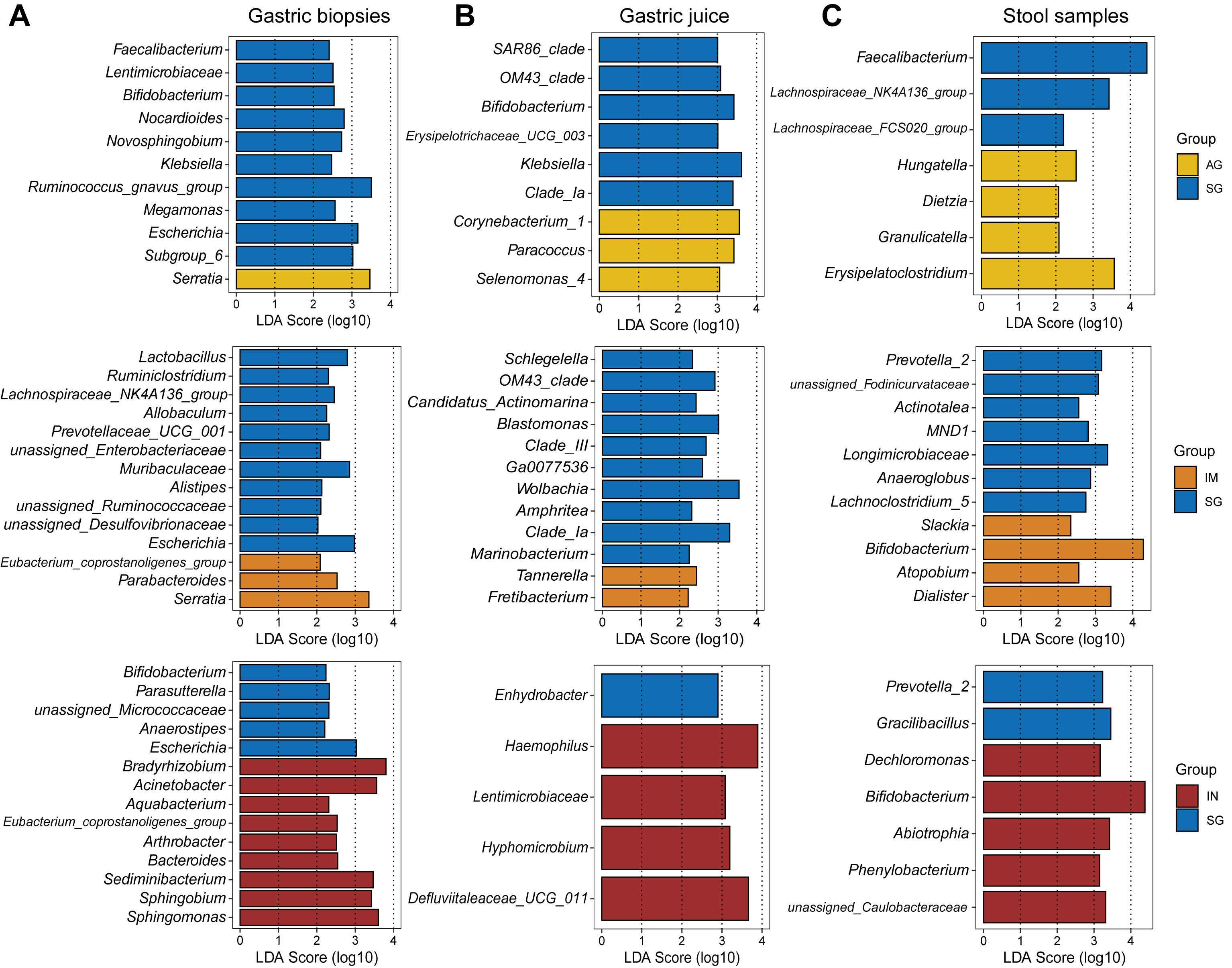


**Figure S****10.** LEfSe comparing the superficial gastritis and gastric precancerous lesions subgroups in self-sequenced dataset. LEfSe test at the genus level comparing (A) the SG group vs AG/IM/IN subgroup in gastric biopsies, (B) the SG group vs AG/IM/IN subgroup in gastric juices, and (C) the SG group vs AG/IM/IN subgroup in stool samples.


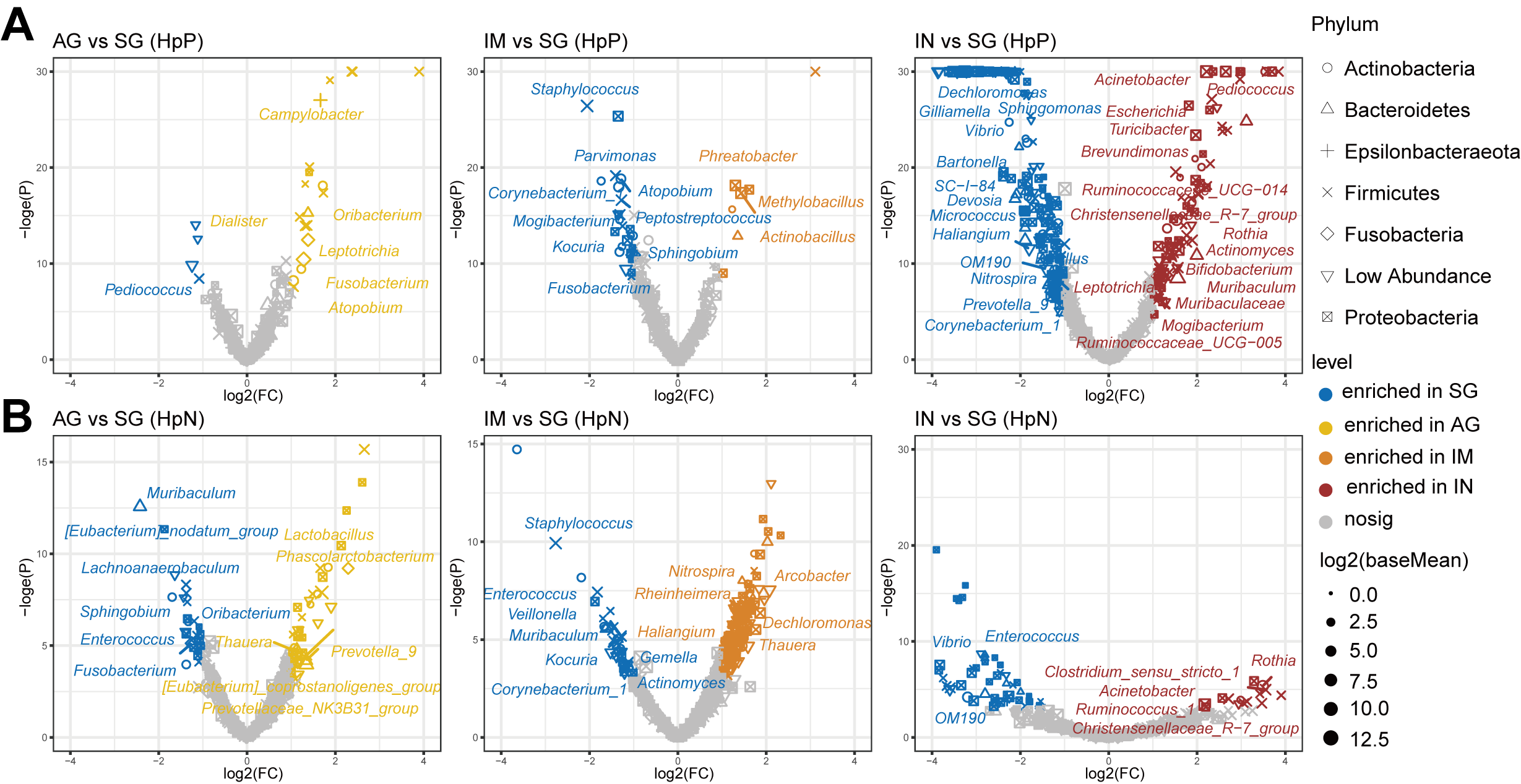


**Figure S****11**. Specific bacterial taxa associated with PLGC in validation datasets. (A) Significantly changed mucosal bacteria in different PLGC subgroups of *H. pylori*-positive subjects and (B) *H. pylori*-negative subjects.
